# Computational evaluation and benchmark study of 342 crystallographic holo-structures of SARS-CoV-2 Mpro enzyme

**DOI:** 10.1038/s41598-024-65228-5

**Published:** 2024-06-20

**Authors:** Hamlet Khachatryan, Mher Matevosyan, Vardan Harutyunyan, Smbat Gevorgyan, Anastasiya Shavina, Irina Tirosyan, Yeva Gabrielyan, Marusya Ayvazyan, Marine Bozdaganyan, Zeynab Fakhar, Sajjad Gharaghani, Hovakim Zakaryan

**Affiliations:** 1 Denovo Sciences Inc, 0060 Yerevan, Armenia; 2 Laboratory of Antiviral Drug Discovery, Institute of Molecular Biology of NAS, Hasratyan 7, 0014 Yerevan, Armenia; 3 Laboratory of Bioinformatics and Drug Design (LBD), Institute of Biochemistry and Biophysics, University of Tehran, Tehran, Iran

**Keywords:** Coronavirus, COVID-19, Mpro, Structural clustering, Virtual screening, Conformational analysis, Protein structure predictions, Virtual drug screening, Antivirals, SARS-CoV-2

## Abstract

The coronavirus disease 19 pandemic, caused by severe acute respiratory syndrome coronavirus 2 (SARS-CoV-2), has led to a global health crisis with millions of confirmed cases and related deaths. The main protease (Mpro) of SARS-CoV-2 is crucial for viral replication and presents an attractive target for drug development. Despite the approval of some drugs, the search for effective treatments continues. In this study, we systematically evaluated 342 holo-crystal structures of Mpro to identify optimal conformations for structure-based virtual screening (SBVS). Our analysis revealed limited structural flexibility among the structures. Three docking programs, AutoDock Vina, rDock, and Glide were employed to assess the efficiency of virtual screening, revealing diverse performances across selected Mpro structures. We found that the structures 5RHE, 7DDC, and 7DPU (PDB Ids) consistently displayed the lowest EF, AUC, and BEDROCK scores. Furthermore, these structures demonstrated the worst pose prediction results in all docking programs. Two structural differences contribute to variations in docking performance: the absence of the S1 subsite in 7DDC and 7DPU, and the presence of a subpocket in the S2 subsite of 7DDC, 7DPU, and 5RHE. These findings underscore the importance of selecting appropriate Mpro conformations for SBVS, providing valuable insights for advancing drug discovery efforts.

## Introduction

Since the first case of coronavirus disease 19 (COVID-19) reported in December 2019 in Wuhan, China, there have been more than 700 million confirmed cases and over 6.9 million related deaths to date. Severe acute respiratory syndrome coronavirus 2 (SARS-CoV-2) is the causative agent of COVID-19. It is a single-stranded positive-sense RNA virus, which belongs to the subgenus *Sarbecovirus* of beta-coronaviruses^1^. Once SARS-CoV-2 enters the host cells, the translation of viral RNA results in the synthesis of two polyproteins, pp1a (490 kDa) and pp1ab (794 kDa). These polyproteins are processed into 16 nonstructural proteins by the main protease (Mpro) and the papain-like protease (PLpro)^2^. After auto-cleavaging itself from the polyproteins, the mature Mpro, a cysteine protease, forms a functional homodimer that transcleaves pp1a and pp1ab at no less than 11 sites^2,3^.

Considering that Mpro plays an important role in SARS-CoV-2 replication and no homologous proteases have been identified in humans^4^, these facts make Mpro a promising target for COVID-19 treatment development^5^. The Food and Drug Administration (FDA) has approved an oral therapeutic called Paxlovid that can reduce the risk of death by 89%^6^. Paxlovid comprises nirmatrelvir, a SARS-CoV-2 Mpro inhibitor, and ritonavir, which inhibits the CYP3A-mediated metabolism of nirmatrelvir. Despite the efficiency of this and some other drugs, the search for new treatments against SARS-CoV-2 is far from the end. Multiple drug discovery approaches such as high-throughput screening, virtual screening, and drug repurposing have been utilized to discover potent inhibitors of Mpro against SARS-CoV-2 and other human coronaviruses^7–9^. Among these approaches, structure-based virtual screening (SBVS) has been widely applied due to its fast and cost-efficient procedure^10–14^. Additionally, the availability of a substantial number of Protein Data Bank (PDB) structures further enhances the applicability of virtual screening. As of May 29, 2024, the PDB includes 4291 protein structures of SARS-CoV-2, with 1374 of these specifically detailing Mpro at a resolution of less than 2.5 Å. These Mpro structures not only offer potential suitability for SBVS but also contribute to the development of pharmacophore models^14,15^.

We have previously demonstrated that the performance of SBVS could be significantly improved depending on the conformation of the target annotated in PDB^16^. In the case of Mpro, the ever-growing number of available PDB structures highlights the need to properly select the most suitable Mpro conformations that could be used in SBVS and computational drug design. In the present study, we systematically assessed a total of 342 holo-structures of Mpro of SARS-CoV-2, undertaking comprehensive benchmark analyses through the utilization of both open-source and commercially available docking software. While Mpro functions as a dimer, we performed all our computational experiments in its monomer form, thus saving time on computational calculations. Although our studies reveal that the examined structures exhibit limited flexibility, diverse docking programs exhibited a range of performances depending on the Mpro structures. Consequently, based on our acquired insights, we advocate for the selection of optimal structures to enhance binding pose prediction accuracy and to facilitate effective virtual screening across diverse docking programs.

## Results

### Extraction of Mpro structures and ligands

**Figure 1:**
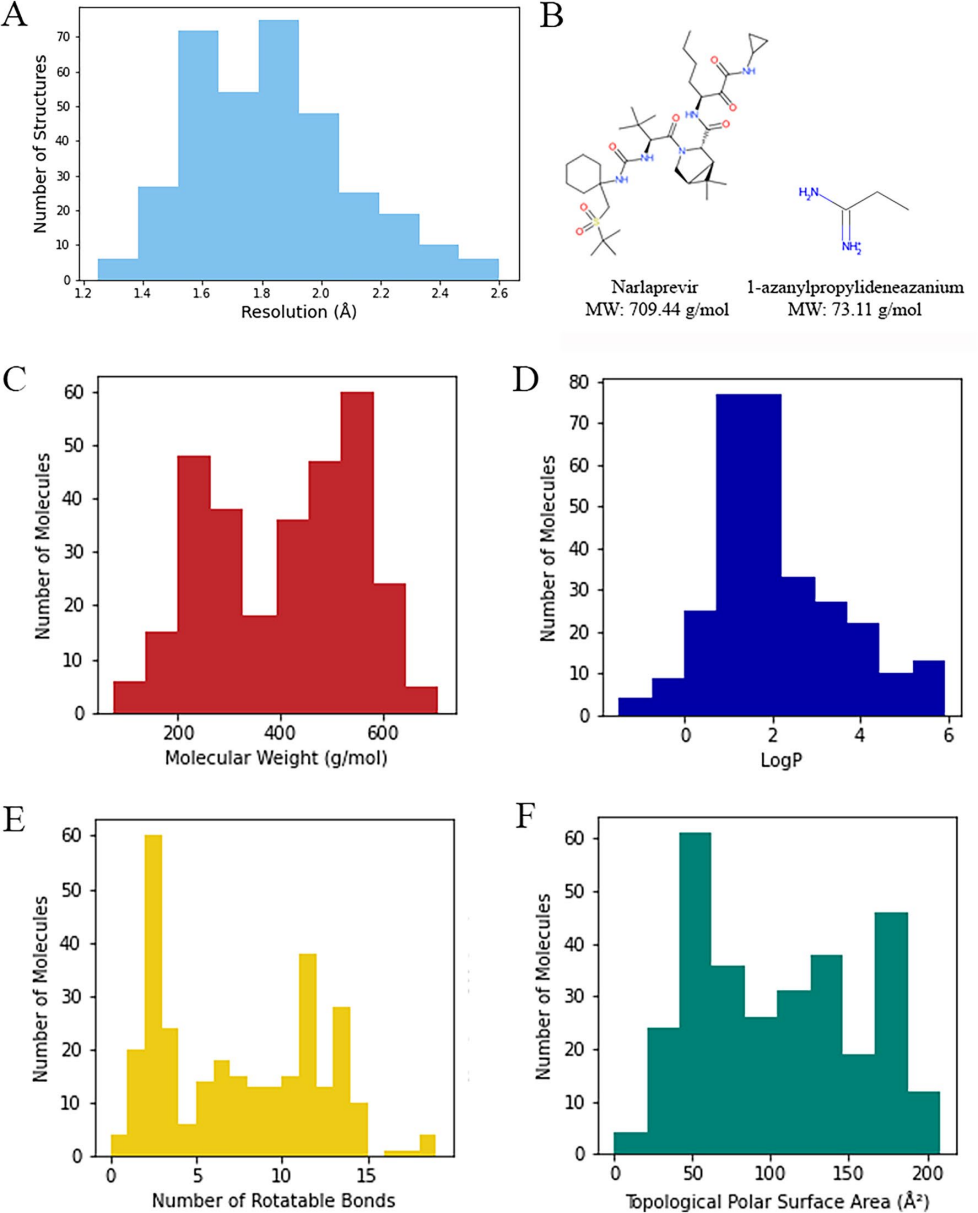
Distribution of holo-structures and ligands. **(A)** Histogram depicting the distribution of holo-structures based on their crystallographic resolutions, expressed in angstroms (Å). **(B)** Chemical structures of the largest and smallest ligands extracted from 342 PDB entries of Mpro. Histograms illustrate the distribution of extracted ligands according to their molecular weight **(C)**, logP **(D)**, rotatable bonds **(E)**, and topological polar surface area properties **(F)**.

As the ligand-bound (holo) structures usually outperform ligand-free (apo) structures in SBVS^17,18^, here we focused on the holo-structures of SARS-CoV-2 Mpro. We identified 454 holo-structures with resolution ≤ 2.5 Å annotated in PDB by 1st November 2022. Then, 112 structures were filtered out as they contained missing amino acid residues, single atom ligands (Se or Au), oligopeptides or ligands located outside of Mpro catalytic (active) site. Most of the remaining 342 structures had resolution ≤ 2.0 Å, including 7JKV (PDB Id) and 5R8T with the highest resolutions 1.25 Å and 1.27 Å, respectively (Fig. 1A, Dataset S1). From 342 PDB structures, we extracted 297 unique ligands, which are diverse and represent many different classes of molecules. The largest ligand was narlaprevir (709.44 g/mol), which covalently bound to 6XQT, 7D1O and 7JYC structures. The smallest ligand was 1-azanylpropylideneazanium (73.11 g/mol), which was found in 5RF2 structure (Fig. 1B). The average molecular weight of the ligands was 408.73 g/mol with 63.3% of ligands less than 500 g/mol (Fig. 1C). Most ligands could be predicted to have a good oral bioavailability as they displayed LogP less than 5, number of rotatable bonds less than 10 and TPSA (Topological Polar Surface Area) less than 150 Å (Fig. 1D, 1E, 1F, Dataset S2)^19^.

### Interacting amino acid distribution

Next, we determined the distribution of amino acid residues involved in the interaction with extracted 297 ligands at the catalytic site of Mpro. As shown in Fig. S1A, more than 50% of ligands interacted with His41, Gly143, Cys145, Glu166 and Gln189 residues, which are among the key residues in the catalytic site of Mpro (Fig. S1B). His41 and Cys145 residues are located at the S1′ subsite of the binding pocket and form the catalytic dyad (Fig. S1B). The 63% evaluated ligands (187 from 297) formed covalent bonds with Cys145. Their capability to form a covalent bond depends on different electrophilic warheads, which are susceptible to the nucleophilic addition of the cysteine-SH. For instance, nirmatrelvir (PF-07321332, PDB Id: 4WI) contains a nitrile warhead, which can form a reversible covalent thioimidate adduct with the sulfur atom of the Cys145 (Fig. S1C)^20^. Other 110 studied ligands such as ML188 (PDB Id: 0EN) could not form covalent bonds with Cys145 due to the absence of electrophilic warheads close to the sulfur atom of the Cys145 (Fig. S1D)^21^. Gly143 at the S1′ subpocket stabilizes polypeptide substrates or ligands in the catalytic site by forming the so-called oxyanion “hole” with Cys145 (Fig. S1B)^22^. Glu166 and Gln189 are located at S1 and S3/4 subsites, respectively, and interact with different ligands via hydrogen bonds (Fig. S1B). The alpha carbon atoms of identified interacting amino acids were used for superimposition and further structural clusterization of 342 PDB structures.

### Structural clusterization and flexibility of Mpro

**Figure 2:**
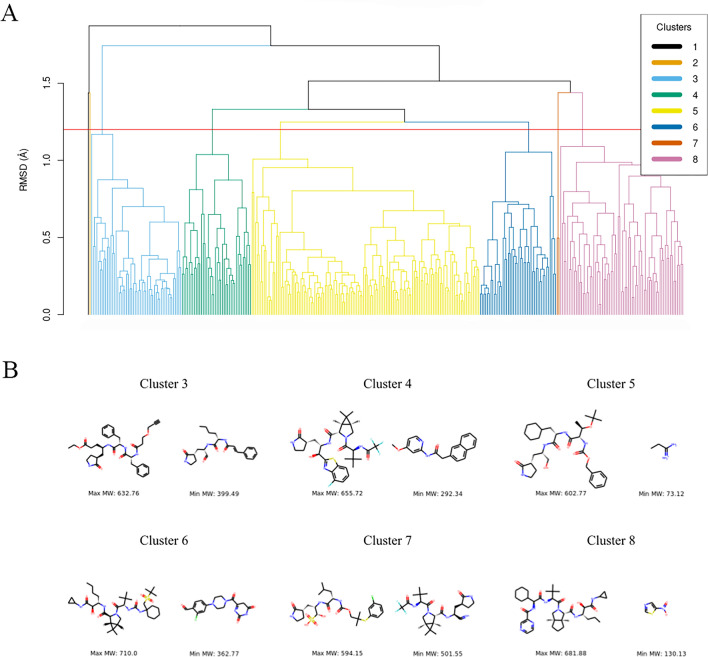
Dendrogram of 342 holo-structures of Mpro based on alpha-carbon atom coordinates of interacting amino acids **(A)**. The red line represents the threshold (1.2 Å) in the RMSD used to identify clusters. The chemical diversity of ligands within each cluster is depicted in **(B)**. Clusters 1 and 2 are not presented, as each cluster consists of only one Mpro structure.

To reduce the computational costs associated with studying 342 PDB structures, we superimposed and clusterized them based on the alpha-carbon atom coordinates of interacting amino acids^16^. Using a 1.2 Å threshold in the Root Mean Square Deviation (RMSD) dendrogram (Fig. 2A), we identified eight clusters. Clusters 1 and 2 were represented by one crystal structure each, while the largest clusters, 5 and 8, contained 131 and 71 structures, respectively. Interestingly, a visual inspection of ligands in holo-structures revealed chemical diversity within each cluster (Fig. 2B, Table S1). To verify this observation, we calculated the average Tanimoto similarity of all unique ligands in each cluster. As shown in table S1, the largest cluster had the lowest similarity value (0.199) between ligands. Four Mpro structures containing the same ligand (PDB Id: 4WI) were found in four different clusters, suggesting that Mpro may undergo different conformational changes after binding with the same ligand.

To select representative Mpro structures, we constructed model structures for each cluster, except for clusters 1 and 2, by averaging the coordinates of the alpha carbon atoms of interacting amino acids of cluster structures. The representative structures were chosen based on the lowest RMSD when comparing the model structure to the alpha carbons of the interacting amino acids of the proteins within each cluster. The structures 7T43, 7RFS, 5RHE, 7TE0, 7VLP, and 6WNP (PDB Ids) were selected as representatives of clusters 3 to 8, respectively (Table S1). These structures with 7DDC and 7DPU from clusters 1 and 2, correspondingly, were used to evaluate the flexibility of Mpro by atomic fluctuations and backbone analysis.

**Figure 3:**
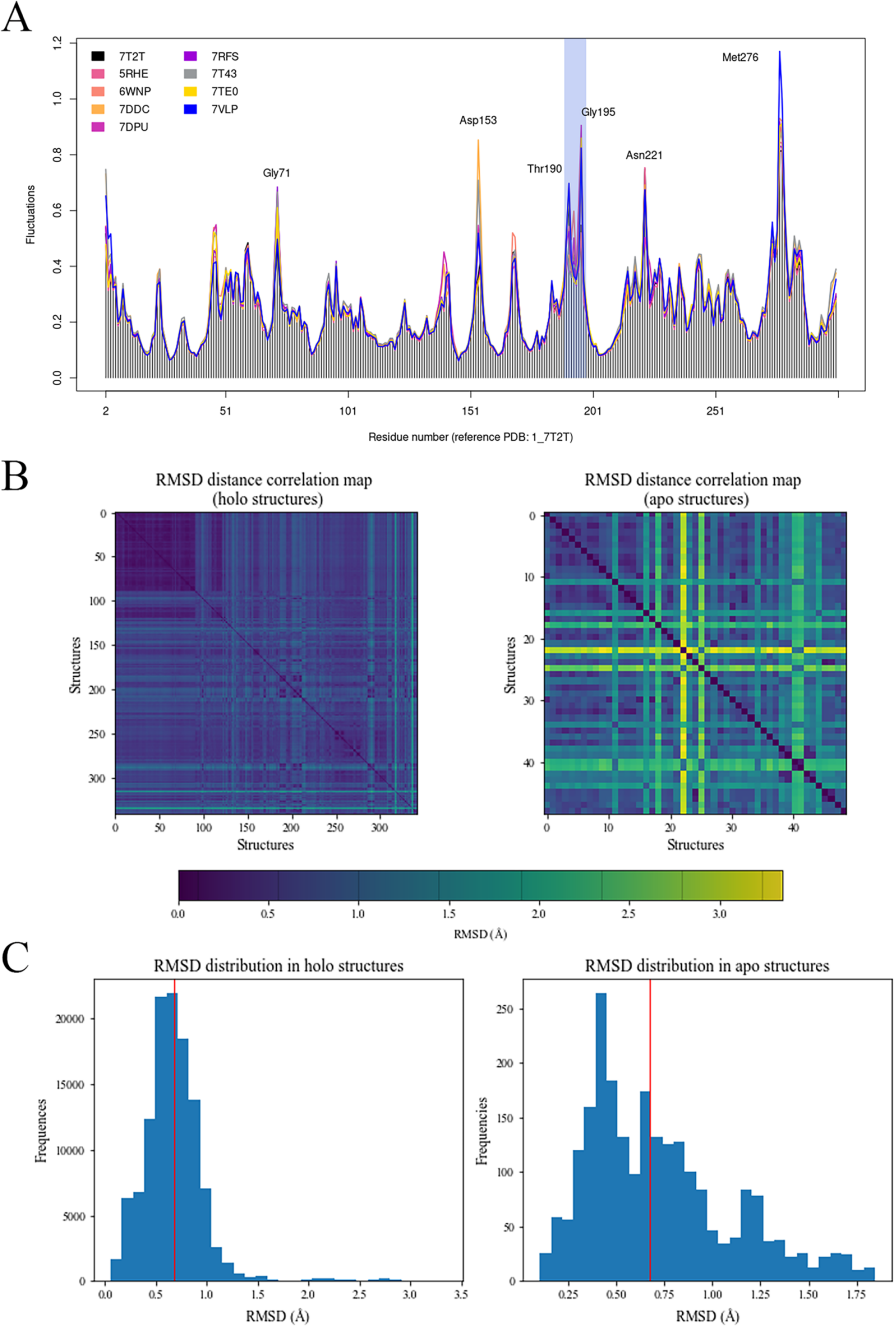
Flexibility of Mpro backbone. **(A)** Results of ensemble NMA of eight studied structures, annotated with labels of amino acids with high fluctuations. The main differences in patterns of fluctuations highlighted with light blue. **(B)** RMSD distance heatmaps for the holo and apo structures with the colormap expressed in angstroms (Å). **(C)** Distributions of the RMSD values within holo and apo structures expressed in angstroms (Å). The red lines represent the means of the distributions.

Next, we performed an elastic network model-based normal mode analysis (NMA) to calculate atomic fluctuations. NMA is a widely used simulation method to probe the functional motions of proteins. Fluctuations for each amino acid were determined by calculating the displacement vectors of atoms for individual normal modes. As shown in Fig. 3A, we found moderate fluctuations (0.6–1 Å) in six regions, which mostly overlapped with the disordered parts of Mpro that were evaluated by IUPred2 (Fig. S1)^23,24^. The amino acid residues with the highest levels of fluctuation were Gly71, Asp153, Thr190, Gly195, Asn221, and Met276 (Table S2). In contrast, the amino acid residues with the lowest levels of fluctuation were His41, Gly143, and Cys145 (Table S1). The main difference in patterns was observed in the region between Thr190 and Gly195 amino acids (light blue region in Fig. 3A), which corresponds to the unstructured part in the II domain (marked with red arrow in Fig. S1B). To compare conformational diversity between holo- and apo-structures we also calculated the mean RMSD values of Cα backbones of 342 holo- and 49 apo-structures. The correlation plots were generated using RMSD distance matrices for the holo and apo structures (Fig. 3B, Dataset S3). The analysis indicated that the apo structures exhibit a slightly higher level of flexibility compared to the holo-structures, supporting findings reported by Clark et al.^25^. This distinction was further emphasized by the RMSD distribution histograms (Fig. 3C), where the mean values are represented by the red lines (0.707 Å and 0.671 Å for apo- and holo-structures, respectively). Based on these results we conclude that the observed structures exhibit limited structural flexibility which is consistent with the observed fact that all scores (probability of being part of a disordered region) of nonterminal amino acids predicted by the IUPred2 long model were lower than 0.5 (maximum score ~ 0.4) (Fig. S2).

### Efficiency of virtual screening for selected Mpro structures

**Figure 4:**
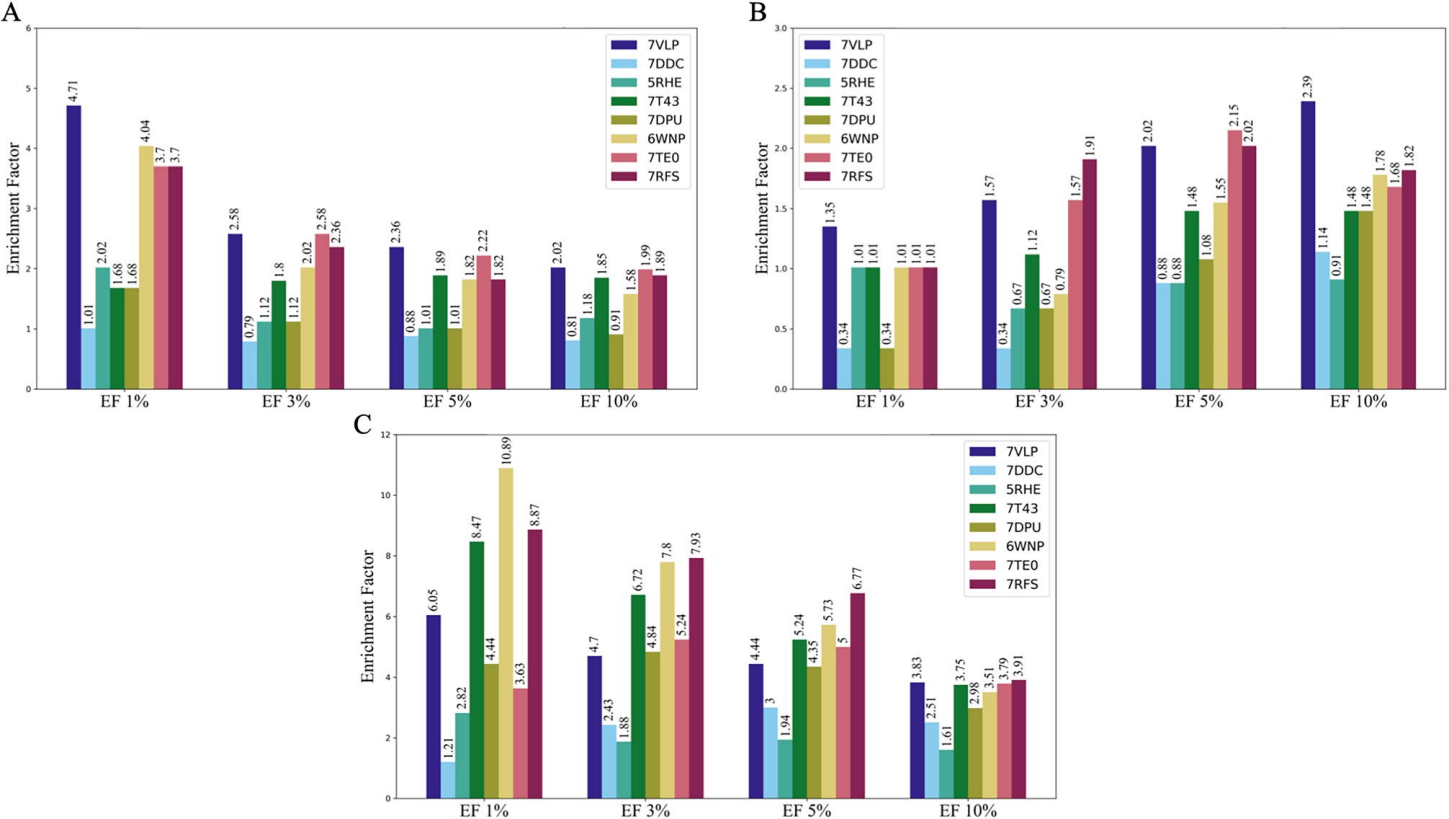
Evaluation of docking performance using AutoDock Vina **(A)**, rDock **(B)**, and Glide **(C)** on selected Mpro structures assessed through Enrichment Factor analysis.

Given the minimal flexibility and conformational differences among annotated Mpro structures, we sought to study the sensitivity of selected PDB structures from all clusters to the virtual screening process. Our objective was to determine if certain structures exhibit enhanced sensitivity to molecular docking compared to others. For this purpose, we employed a dataset consisting of 297 unique active ligands extracted from 342 PDB structures (Dataset S2). Additionally, we included 8910 decoys (Dataset S4) in our analysis resulting in an overall 9207 molecules. Molecular docking simulations were performed using three different software: AutoDock Vina and RDock, which are open-source programs, as well as Glide, which is a commercially available program. Different molecular docking software exhibited varying performances when applied to the same PDB structures, which is consistent with previously published results^26^ (Fig. 4). When evaluating virtual screening, we primarily used three widely studied metrics: Enrichment Factors (EF), Area Under the Receiver Operating Characteristic Curve (ROC-AUC), and Boltzmann-Enhanced Discrimination of ROC (BEDROC). These are extensively used for ranking method evaluations. Interpretation and calculation details of the above scores are presented in the 4.2.6 section.

RDock exhibited the poorest performance, displaying the lowest enrichment factor (EF) for all eight representative PDB structures (Fig. 4B). AutoDock Vina demonstrated slightly improved EFs (Fig. 4A). In contrast, Glide showed significantly higher EFs across at least five distinct PDB structures (Fig. 4C). Interestingly, the structure 7DDC, representing cluster 2, consistently displayed the lowest EFs across all utilized docking programs. Both RDock and AutoDock Vina exhibited their highest EFs when the structure 7VLP was employed. In addition, the highest EFs for Glide were observed for the structures 7RFS and 6WNP, representing clusters 4 and 8, respectively.

The ROC-AUC analysis revealed similar results for the selected PDB structures. Notably, both 7DDC and 5RHE structures exhibited the lowest AUC values for all docking programs, as shown in Fig. S3–S5. The screening using Glide yielded the highest AUC values, particularly for the structures 7T43, 7RFS, and 7VLP, with values of 0.717, 0.707, and 0.691, respectively (Fig. S5). We observed similar trends of performance of selected PDB structures for the BEDROCK scores with alpha value equal to 20, with small differences in rankings of best-performing structures compared to enrichment factors (Fig. S6). However, the worst-performing structures evaluated by this metric are the same as in evaluations with EF and ROC-AUC scores.

### Efficiency of binding pose prediction for virtual screening

**Figure 5:**
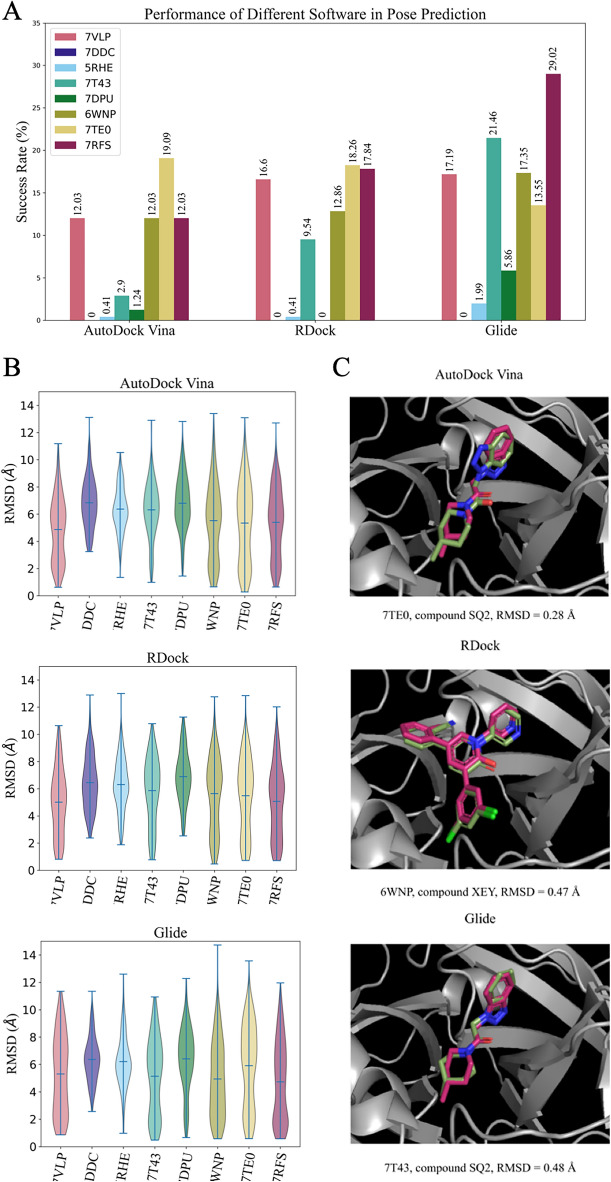
Ligand pose prediction performance on selected Mpro structures. **(A)** Assessment of pose prediction accuracy for selected Mpro structures. The success rate indicates the proportion of docked poses with RMSD less than 2 Å compared to the same ligands in the corresponding crystallographic structures. **(B)** Distribution of poses by RMSD values for each selected Mpro structure. **(C)** The best-predicted poses with their corresponding crystallographic structures. Green—docked pose; pink—native crystallographic pose.

To investigate whether the performance of binding pose prediction depends on the crystallographic structures, we analyzed the RMSD between the docked poses of active molecules and their corresponding X-ray crystallographic structures. Poses exhibiting an RMSD less than 2 Å were considered as an accurate and acceptable docking pose^27,28^. We utilized the “success rate” metric, employing a threshold of 2 Å. This metric evaluates the proportion of poses with an RMSD below 2 Å in comparison to the crystallographic structures. The success rates for pose prediction demonstrated performance trends similar to those observed in virtual screening performance. Consistently bad pose prediction results were observed for the structures 7DDC, 7DPU, and 5RHE by all docking programs (Fig. 5A). Docking programs were not able to predict accurate poses with an RMSD less than 2 Å for 7DDC. The highest success rate, 29.02%, was observed on the 7RFS structure by the Glide docking program (Fig. 5A). Figure 5B shows the distributions of RMSD values for every docking program and selected structures. The range of distributions is similar to each other with quite close mean values. The worst RMSD was calculated by Glide for the 6WNP structure with a value higher than 15 Å (Fig. 5B). In the case of good-performing structures, 7VLP, 7T43, 6WNP, 7TE0, and 7RFS, the bimodal-like distributions were observed (except 7T43 for AutoDock Vina) suggesting that there could be a pool of ligands whose docked poses demonstrating better sensitivity to studied PDB structures. However, structural analysis of active compounds with accurate docked poses for best-performing software and protein (Glide and 7RFS structure) demonstrated that mean Tanimoto similarity within docked ligands with RMSD less or equal to 2 Å is 0.36, which is comparable to mean Tanimoto similarity within docked ligands with RMSD more than 2 Å: 0.24 (Fig. S7). The best-predicted poses with their corresponding crystallographic structures for every studied software are shown in Fig. 5C.

### Conformational comparison of selected Mpro structures

**Figure 6:**
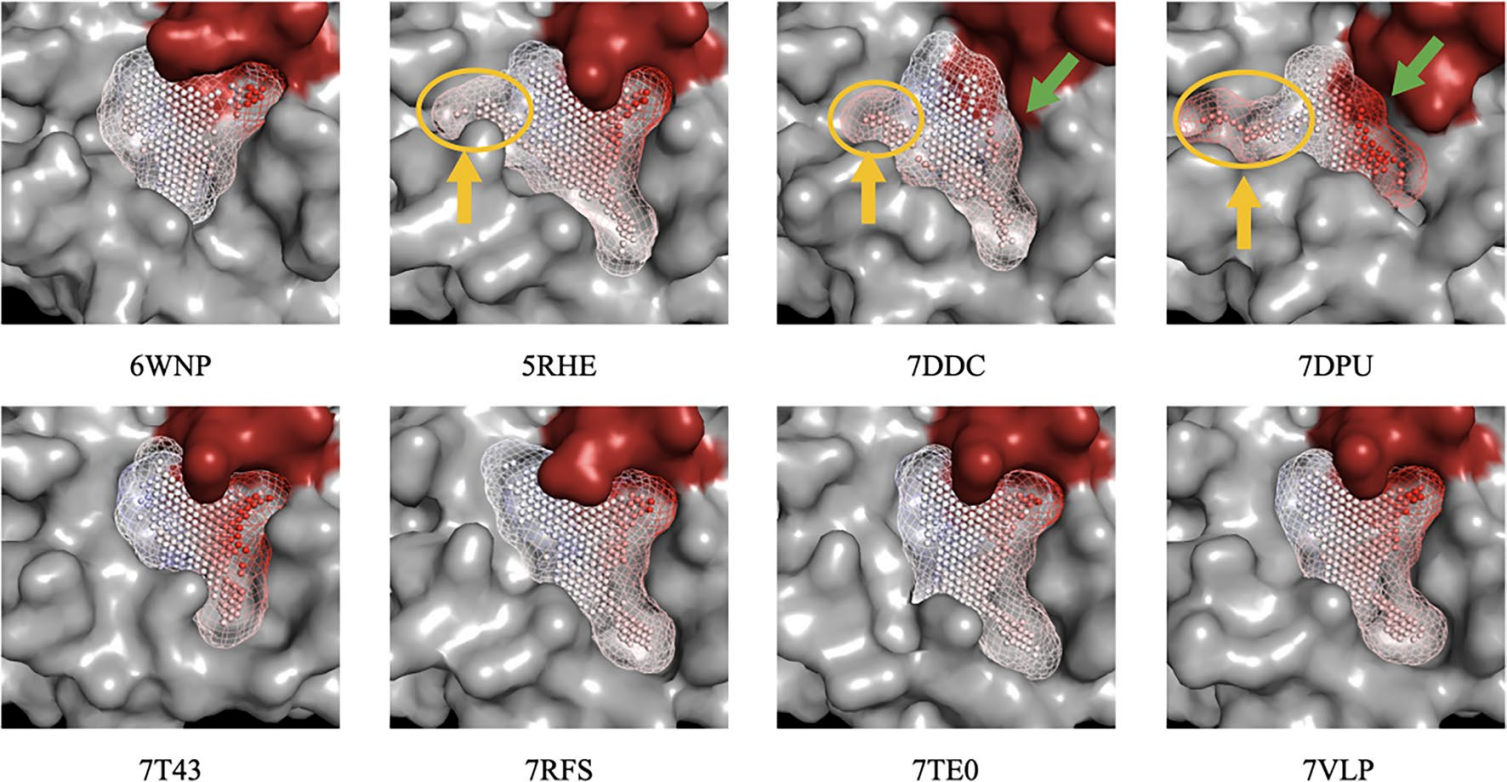
Binding sites of selected Mpro structures. Binding sites are represented as point clouds filling the binding cavity, annotated by electrostatic potential values at each point. The red surface in the cavity indicates Cys145. The absence of the S1 subsite in 7DDC and 7DPU, and the existence of a subpocket in the S2 subsite of 7DDC, 7DPU, and 5RHE were highlighted by green and yellow arrows, respectively.

To understand the variance in performance metrics among the examined PDB structures, we first visualized the binding sites of selected structures as annotated point clouds filling the cavity. Electrostatic potentials were used for each point as an annotation. The visual analysis of cavities revealed mainly two structural differences between the best and worst structures: the absence of the S1 subsite in 7DDC and 7DPU, and the existence of a subpocket in the S2 subsite of 7DDC, 7DPU, and 5RHE (Fig. 6).

**Figure 7:**
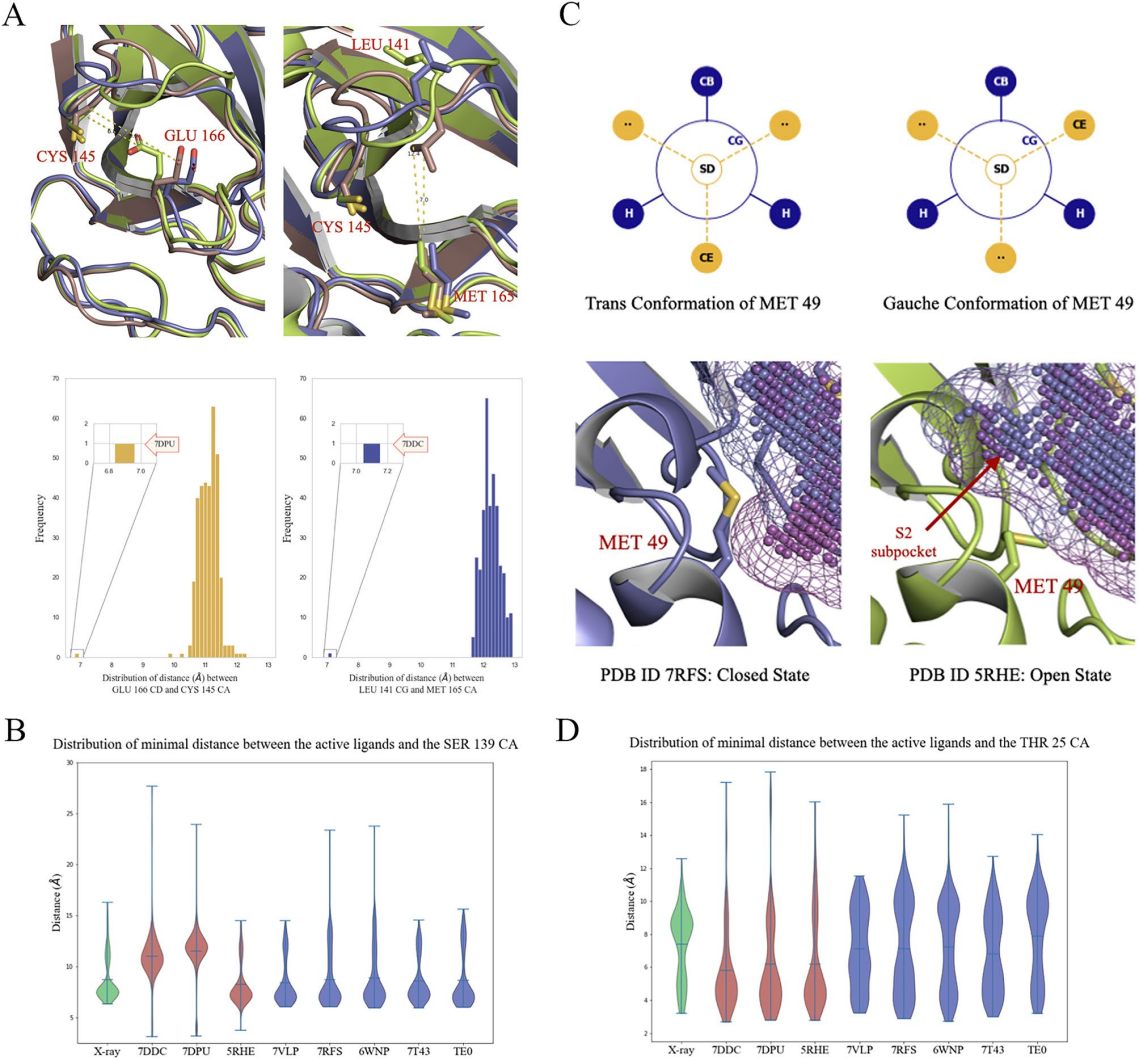
Conformational flexibility of binding site residues within studied structures. **(A)** Top left and right figures show the conformational flips of amino acids resulting in the absence of S1 subsite in 7DPU and 7DDC structures, respectively (blue—7RFS, green—7DPU, brown—7DDC). Bottom is the distributions of distances between Glu166 CD—Cys145 CA and Leu141 CG—Met165 CA, expressed in angstroms (Å) among studied crystallographic structures **(B)** Violin plot of the Probability Density Function (PDF) distributions of shortest distances between active ligands and the alpha carbon of Ser139 across all crystallographic and docked structures, expressed in angstroms (Å). **(C)** Trans and gauche conformations of the Met49 amino acids and corresponding closed (7RFS) and open (5RHE) states. **(D)** Violin plot of the PDF distributions of shortest distances between active ligands and the alpha carbon of Thr25 across all crystallographic and docked structures, expressed in angstroms (Å).

Upon further conformational analysis, we revealed that the absence of the S1 subsite in 7DDC and 7DPU was due to the flipping of Leu141 and Glu166 side chains, respectively (Fig. 7A). Such side chain flipping phenomena were not observed in other structural conformations. To assess the impact of the S1 subsite on virtual screening efficacy, we analyzed the shortest distances between active ligands and the alpha carbon of Ser139, an amino acid residue located at the S1 subsite across all crystallographic and docked structures. We hypothesized that if active ligands occupied the S1 subsite, the distance would be shorter compared to docked ligands in 7DDC and 7DPU. As expected, the mean distances in the distributions of the two underperforming structures, 7DDC and 7DPU, which lack the S1 pocket, were significantly higher than those observed in other structures (Fig. 7B). Moreover, the mean and median distribution of distances in crystallographic structures aligned closely with those in all other structures except 7DDC and 7DPU, indicating that the existence of the S1 subsite within the PDB structures is likely to exert a crucial influence on the effectiveness of virtual screening and pose prediction performances.

The presence of a subpocket within the S2 subsite is primarily attributed to the flexibility exhibited by Met49, acting like a “gate.” In the closed state, where the subpocket does not exist, Met49 adopts a trans conformation, which is energetically more favorable than the gauche conformation assumed in the open state when the subpocket is present (Fig. 7C). Subsequently, we computed the shortest distances between active ligands and the Thr25 alpha carbon located at the subpocket. We hypothesized that inaccuracies in the poses of active ligands in structures 7DDC, 7DPU, and 5RHE could be linked to ligands occupying this subpocket. The findings revealed that the majority of docked ligands were positioned in closer proximity to Thr25 for the structures with subpockets (7DDC, 7DPU, and 5RHE) (Fig. 7D). In contrast, other structures exhibited distributions similar to those observed in crystal structures, emphasizing the role of the absence of this subpocket for successful virtual screening.

## Discussion

In the context of drug discovery and virtual screening, benchmark studies traditionally focus on evaluating the performance of docking software^29^, often overlooking the critical aspect of benchmarking PDB structures. Several metrics, such as R-value, Clashscore, Ramachandran, and Real-Space R-value Z score (RSRZ) outliers, should be considered when selecting appropriate PDB structures. These scores mainly describe the refinement and diffraction quality of the experimental model and crystal, respectively. Our previous studies have shown that different docking software performances may vary even with PDB structures with similar validation statistics^16^. The discrepancy between the validation scores and SBVS performance can be explained by unfavorable side-chain conformations in a binding pocket/interacting surface, which may fit well into electron density maps. This observation also appeared in this study, emphasizing the importance of selecting the proper protein structure for each docking software.

In the current work, we evaluated 342 holo-structures of SARS-CoV-2 Mpro, which was the maximum number of Mpro structures with a resolution of ≤ 2.5 Å annotated in the PDB database at the moment of initiation of this study. This enzyme emerges as an optimal antiviral target for therapeutic development for two reasons: (i) the absence of homologous protein enzymes in humans, and (ii) its relatively conserved structure among pathogenic β-coronaviruses^30^.

Although Mpro demonstrated structural plasticity between low-temperature ligand-free and inhibitor-bound structures^31^, the mean RMSD values of Cα backbones of 342 holo-structures and normal mode analysis of atomic fluctuations showed low flexibility. We expected that the low flexibility of the binding pocket could minimize the impact of structural variations on docking simulations. To test this hypothesis, we performed molecular docking by using different docking programs and active/decoy compounds. Docking 9207 compounds (297/8910) with 342 holo-structures by three docking programs is time-consuming. We clustered all PDB structures using the alpha-carbon atom coordinates of interacting amino acids. Then, we selected a representative structure from each cluster, significantly reducing computational costs. The subsequent assessment of virtual screening efficiency across different docking programs revealed considerable variability in performance depending on the PDB structure. The structures 5RHE, 7DDC, and 7DPU consistently displayed the lowest EFs in all docking programs. This observation suggests that the structural characteristics of 5RHE, 7DDC, and 7DPU may pose challenges for accurate binding affinity predictions by different docking software. The ROC-AUC analysis further supported the trends observed in the virtual screening results. Structures 7DDC and 5RHE consistently exhibited the lowest AUC values for all docking programs. The structures that performed poorly in terms of EF and ROC-AUC scores were consistent with those identified by BEDROCK analysis.

We further studied the performance of binding pose prediction for selected Mpro structures. Accurate prediction of binding poses holds significant relevance, particularly in cases where interactions with key amino acids, such as Cys145 in Mpro, play an important role in the inhibitory activity of ligands^32^. The RMSD analysis revealed that the accuracy of pose prediction varied across structures and docking programs. Consistently challenging structures 5RHE, 7DDC, and 7DPU demonstrated poor pose prediction results in all docking programs. The success rate metric, defined by the proportion of poses with an RMSD below 2 Å compared with the crystallographic structures, echoed the trends observed in virtual screening performance.

The structural analysis of the examined PDB structures, particularly focusing on the binding sites, revealed two structural differences, primarily contributing to variations in docking performance: the absence of the S1 subsite in 7DDC and 7DPU, and the presence of a subpocket in the S2 subsite of 7DDC, 7DPU, and 5RHE. It was determined that the absence of the S1 subsite in 7DDC and 7DPU resulted from the flipping of Leu141 and Glu166 side chains, respectively. This flipping phenomenon was not observed in other structural conformations. To assess the impact of the S1 subsite on virtual screening efficacy, we analyzed the distances between active ligands and the alpha carbon of Ser139, a key amino acid residue at the S1 subsite. The results indicated that the underperforming structures, 7DDC and 7DPU, lacking the S1 pocket, exhibited significantly higher mean distances compared to other structures. This suggests that the presence of the S1 subsite is crucial for the effectiveness of virtual screening. Indeed, numerous inhibitors, such as the COVID-19 drugs Ensitrelvir and Nirmatrelvir, effectively target the S1 subsite^33,34^. Mutations occurring within this subsite have been identified as contributors to the development of drug resistance^34^, underscoring the critical significance of interactions with amino acids located at the S1 subsite.

The presence of a subpocket within the S2 subsite was linked to the flexibility exhibited by Met49. Analysis of the shortest distances between active ligands and the Thr25 alpha carbon, located at the subpocket, revealed that inaccuracies in ligand poses in structures 7DDC, 7DPU, and 5RHE were associated with ligands occupying this subpocket. Other structures without subpockets exhibited distributions similar to crystal structures, underscoring the importance of the absence of the subpocket for successful virtual screening.

Mpro functions as a homodimer, with each chain playing a crucial role in forming the binding pocket on the other chain. While the structures deposited in the PDB are mostly dimers, we only used the A chains of structures for analysis, which is a limitation of this study. However, our analysis of interacting amino acids showed that only 2.92% of the analyzed structures had interactions between a ligand from chain A and the SER 1 amino acid from chain B. Therefore, the bias in this study is negligible.

In conclusion, our comprehensive analysis provides valuable insights into the structural features influencing the performance of virtual screening in targeting SARS-CoV-2 Mpro. Based on our results, several suggestions for improving virtual screening can be proposed. Firstly, researchers should prioritize benchmarking studies that not only assess docking software performance but also rigorously evaluate the PDB structures used. Secondly, special attention should be given to structures with unique characteristics, such as the absence or presence of key subpockets, as these can significantly impact virtual screening outcomes. Incorporating these considerations into future studies will enhance the performance of virtual screening approaches in drug discovery, particularly for antiviral targets like SARS-CoV-2 Mpro. As the number of new PDB structures of Mpro continues to grow, conducting similar studies on functional dimers of these newly available PDB structures is crucial.

## Materials and methods

### PDB structures clusterization and flexibility analysis

#### Structure selection, filtration and preparation

Structures of the SARS-CoV-2 main protease (resolution ⩽ 2.5 Å) were collected from the PDB (https://www.rcsb.org/) and filtered from structures, which contain single atom ligands (Se or Au), oligopeptide ligands, ligands located outside of binding site and structures without ligands. Structures that do not have PDB files or have missing residues in the binding site were also removed (Dataset S1). Water molecules and ligands were removed from filtered structures, and A chains of structures were selected for further analysis. Cleaned and processed structures were aligned via PyMOL version 2.0 (https://pymol.org/)^35^.

#### Analysis of the physicochemical properties of extracted ligands

Molecular weight, count of rotatable bonds, and TPSA of extracted ligands were calculated using the RDKit open-source cheminformatics toolkit version 2023.03.2 (https://www.rdkit.org/)^36^. Water/octanol partition coefficients (LogP) were calculated using the RDKit implementation of the atom-based calculation approach described by Wildman and Crippen^37^.

#### Clusterization of Mpro structures

Superimposition and hierarchical clusterization were performed based on alpha carbon atom coordinates of interacting amino acids via Bio3D R package (http://thegrantlab.org/bio3d/)^38^ with 1.2 Å cut-off, as described by Chilingaryan et al.^16^. Interacting amino acids were determined via the Protein–Ligand Interaction Profiler (PLIP) tool version 2.3.0 (https://plip-tool.biotec.tu-dresden.de/)^39^. For each cluster, the model structure was created, by averaging the coordinates of alpha carbon atoms of interacting amino acids, and compared with the cluster’s PDB structures. As a representative of each cluster was the structure with the lowest RMSD value compared to the cluster’s average model protein.

#### Backbone analysis

The Normal Mode Analysis (NMA) of selected representative structures was done based on the Elastic Network Model, using the Bio3D R package implemented by Skjærven et al.^40^.

To identify and evaluate disordered regions of protein IUPred2A web interface (https://iupred2a.elte.hu/)^23,24^ was used. Scores (from 0 to 1) assigned to each amino acid by the IUPred2A default model evaluating the probability of localization of amino acid in disordered region: higher score, higher probability of being in disordered region.

The RMSD distance matrix for the holo and apo structures was calculated within selected holo-structures and compared with the RMSD matrix calculated for 49 apo structures (Dataset S3). Apo structures were selected from the PDB based on 100% sequence similarity compared to holo-structures (resolution ⩽ 2.5 Å, except 7CAM structure). Superimposition of structures for RMSD matrix calculation was done based on the most invariant regions of proteins via Bio3D R package^38^.

### Virtual screening: implementation and evaluation

#### Benchmark dataset for virtual screening evaluation

297 unique co-crystallized ligands (Dataset S2) extracted from 342 analyzed structures were used as the dataset of active ligands. The Decoy set (Dataset S4) was generated by the Directory of Useful Decoys: Enhanced^41^ (DUD-E) (https://dude.docking.org/) with a 1/30 ratio to active ligands (8910 unique molecules). A combined dataset containing 9207 compounds was used as a benchmark set for virtual screening experiments.

#### Docking software

For virtual screening experiments, two open-source (AutoDock Vina^42^ version 1.2.3 (https://vina.scripps.edu/) and Rdock^43^ version 0.22 (https://rdock.github.io/)) and one commercially available (Glide^44^) (https://www.schrodinger.com/platform/products/glide/), docking software were used. Selection of software is made based on availability for us and the fact that they are commonly used, ho wever, any other docking software could be used for the same protocol. The main two varying components of docking software are the binding pose search algorithm and the scoring function. For the local optimization and generation of docking poses AutoDock Vina uses an “Iterated Local Search global optimizer” and Broyden–Fletcher–Goldfarb–Shanno local optimization method alongside hybrid, empirical, and knowledge-based, scoring function^42^. The standard docking protocol of RDock consists of three phases (1. Genetic search algorithm 2. Low-temperature Monte Carlo minimization and 3. Simplex minimization) alongside a scoring function based on the combination of the weighted terms of inter- and intra-molecular (ligand, protein/binding site) interaction energies and external restraints. Glide searches for favorable interactions between the ligand and the active site using a filtering approach wherein each of the docked poses passes through a series of hierarchical filters that evaluate the ligand's interaction with the receptor. As a scoring function, Glide uses the Extra Precision^45^ (XP) hybrid scoring function.

#### Preparation of the selected protein structures

This step involves addition of missing hydrogens, the appropriate charge and protonation state assignment (pH = 7.0) to the previously cleaned protein, with consideration of the appropriate ionization states for the acidic and basic residues. For this purpose, AutoDock Tools 4^46^ and UCSF Chimera 1.16 toolkit (https://www.cgl.ucsf.edu/chimera/)^47^ (with ff14SB force field) were used for AutoDock Vina and RDock correspondingly. For Glide, the same procedure was done using its corresponding in-built tools (Protein Preparation Wizard^48^ for Glide). Also, as a part of standard Glide docking protocol, the structures were minimized using OPLS-2005^49,50^ force-field and in-build tools to relieve steric clashes.

#### Preparation of the benchmark molecules

For AutoDock Vina and RDock the conformations of the benchmark ligands were created, protonated, and minimized (force field: MMFF94) using the RDKit cheminformatics toolkit^36^. The preparation of the ligands for Glide was performed using the LigPrep module of Schrodinger Suite which performs the addition of hydrogens and conformation creation. As a part of the standard docking protocol, partial charges were assigned to the structures using the OPLS-2005 force field with further energy minimization.

#### Docking parameters and protocols

To define the binding box, we combined all the active compounds into one molecule as a model ligand after the alignment of corresponding proteins. The binding box for all proteins is defined around this model ligand.

To identify thegrid box for AutoDock Vina, AutoDock Tools version 1.5.7 (https://ccsb.scripps.edu/mgltools/) were utilized. The center of the grid box was located at the center of mass of the model ligand and edges extended to fully surround the binding pocket. The dimensions of the grid were set to size_x: 26 Å, size_y: 25 Å, size_z: 25 Å. The dockings for virtual screening with AutoDockVina were performed according to standard protocol and parameters recommended by the developers with “exhaustiveness” set to 16.

The binding box for RDock was defined around the model ligand via RbtLigandSiteMapper with default parameters and RADIUS = 4.0 angstroms. The binding cavity is mapped by enveloping the volumes of spheres with a radius equal to RADIUS with centers corresponding to atom coordinates of model ligands. The space outside of the overlapped volumes and the space occupied by the protein were excluded. Docking was performed according to the RDock’s general 3-step protocol with 20 runs per ligand.

The binding site grid for Glide was generated as two cubical boxes with a common centroid (the center of mass of the model ligand): a larger enclosing and a smaller binding box with dimensions of 28 × 28 × 28 Å^3^ and 12 × 12 × 12 Å^3^, respectively. Docking was performed using the standard protocol implemented in the Glide workflow of Schrödinger^44,45^. The conformer with the best docking energy/score for each molecule from all software was extracted for further analysis.

#### Virtual screening performance evaluation

To assess the virtual screening performance widely recognizable metrics were used: 1. Enrichment Factors (EF) (for 1, 3, 5 and 10%) 2. Area Under the Receiver Operating Characteristic Curve (ROC-AUC) 3. Boltzmann-Enhanced Discrimination of ROC (BEDROC) with early recognition parameter α = 20. EF and BEDROC metrics were calculated via in-house Python implementation of Truchon and Bayly^51^ work. The EF metric evaluates how many more active compounds are found in an “early recognition” subset (χ) of an ordered list compared to random distribution, and the EF at χ is calculated by the proportion of true active compounds in the selection set relative to the whole dataset:

1 $$EF (\chi ) = \frac{{N}_{hits, \chi }}{{N}_{\chi }} / \frac{{N}_{hits, T}}{{N}_{T}}$$

where ${N}_{hits, \chi }$ and ${N}_{hits, T}$ are number of active compounds in an “early recognition” subset χ and in the total dataset correspondingly, and ${N}_{ \chi }$ and ${N}_{ T}$ are the number of compounds in a subset χ and in the total dataset correspondingly. BEDROCK is an adaptation of the ROC score designed for the “early recognition” task by introducing the exponential of the alpha (a) parameter, which represents the level of “early recognition” needed to weigh the contribution of the rank axis to the final score. Similar to ROC, BEDROC ranges from 0 to 1 and can be understood as the probability that a randomly selected ranked active compound will be positioned before a randomly selected compound distributed according to an exponential of the parameter α. Truchon and Bayly^51^ suggest that α be set to 20, which means that 80% of the maximum contribution to the BEDROC comes from the first 8% of the dataset.

For ROC-AUC calculation the Python Scikit Learn (https://scikit-learn.org/)^52^ library was used. Enrichment Factors at the mentioned thresholds were used as a main metric for virtual screening performance evaluation, however, all metrics were consistent for each analyzed protein structure.

#### Binding pose prediction evaluation

To assess the performance of studied software and structures in the matter of binding pose prediction, the conformation with the best docking score/energy for active ligands was compared with poses from their native X-ray crystallographic structures. To perform this experiment, a cleaned PDB structure of each active ligand was aligned to the studied protein structure via PyMOL. Furtherly RMSD between docked conformation and native binding position of the active ligands were calculated. To evaluate the performance of virtual screening in binding pose prediction, distributions of RMSDs were compared. Also, the commonly used “Success Rate” metric, defined as the proportion of ligands with RMSDs less than 2 Å, was utilized for performance evaluation^53,54^.

#### Conformational analysis of binding pockets of selected Mpro structures

For point cloud-based visualization of binding pockets and annotation of each point within a cloud with electronegativity potentials PyMOL’s CavitOmiX (v. 1.0, 2022, Innophore GmbH) (https://innophore.com/cavitomix/) plugin was used. Conformational flexibility analysis of the binding site residues was performed based on the hypotheses described in section Results 6, using PyMOL for distance calculations and Python for distribution analysis.

To create visual illustrations and graphs, PyMol, R Bio3D package and Python Matplotlib package^55^ were used.

### Supplementary Information

Supplementary Information 1.

Supplementary Information 2.

Supplementary Information 3.

Supplementary Information 4.

Supplementary Information 5.

## Data Availability

The datasets used during the current study are attached as supplementary datasets (Dataset S1, S2, S3, S4). Additional information is available from the corresponding authors on reasonable request.
