# Supplementary material for: Computational evaluation and benchmark study of 342 crystallographic holo-structures of SARS-CoV-2 Mpro enzyme
Source: Sci Rep. 2024 Jun 20;14:14255. doi: 10.1038/s41598-024-65228-5 (PMC11189913; doi:10.1038/s41598-024-65228-5)
Supplement: Supplementary file 5 — Supplementary Information 5. [file 41598_2024_65228_MOESM5_ESM.docx]

Computational evaluation and benchmark study of 342 crystallographic holo-structures of SARS-CoV-2 Mpro enzyme

Hamlet Khachatryan^1,2,*^, Mher Matevosyan^2^, Vardan Harutyunyan^2^, Smbat Gevorgyan^1,2^, Anastasiya Shavina^1,2^, Irina Tirosyan^2^, Yeva Gabrielyan^2^, Marusya Ayvazyan^2^, Marine Bozdaganyan^1^, Zeynab Fakhar^3^, Sajjad Gharaghani^3^, Hovakim Zakaryan^1,2,*^

^1^Laboratory of Antiviral Drug Discovery, Institute of Molecular Biology of NAS, 0014, Hasratyan 7, Yerevan, Armenia.

^2^ Denovo Sciences Inc, 0060, Yerevan, Armenia.

^3^ Laboratory of Bioinformatics and Drug Design (LBD), Institute of Biochemistry and Biophysics, University of Tehran, Tehran, Iran.

*** Corresponding Authors:**

Hovakim Zakaryan, Institute of Molecular Biology of NAS, 0014, Hasratyan 7, Yerevan, Armenia, tel: +37491318036, E-mail: [hovakimz@denovosciences.ai](mailto:hovakimz@denovosciences.ai); [h_zakaryan@mb.sci.am](mailto:h_zakaryan@mb.sci.am)

Hamlet Khachatryan, Institute of Molecular Biology of NAS, 0014, Hasratyan 7, Yerevan, Armenia, tel: +37491318036, E-mail: hamletk@denovosciences.ai

**Supplementary Materials**

The supplementary materials contain two tables, seven figures, and four datasets manually constructed for this research (in CSV format).

**Dataset S1:** CSV file containing information about the analyzed structures: PDB identifier, resolution of crystal structure, corresponding cluster of the structure after structural clustering.

**Dataset S2:** CSV file containing information about the extracted ligands from crystal structures: PDB identifier, canonical smiles representation, molecular weight, number of atoms, number of rotatable bonds, LogP and TPSA (Topological Polar Surface Area).

**Dataset S3:** CSV file containing information about the analyzed apo structures: PDB identifiers and resolutions of corresponding crystal structures.

**Dataset S4:** CSV file containing information about the decoy set generated by DUD-E. The file includes the smiles representations of the decoy ligands.

**
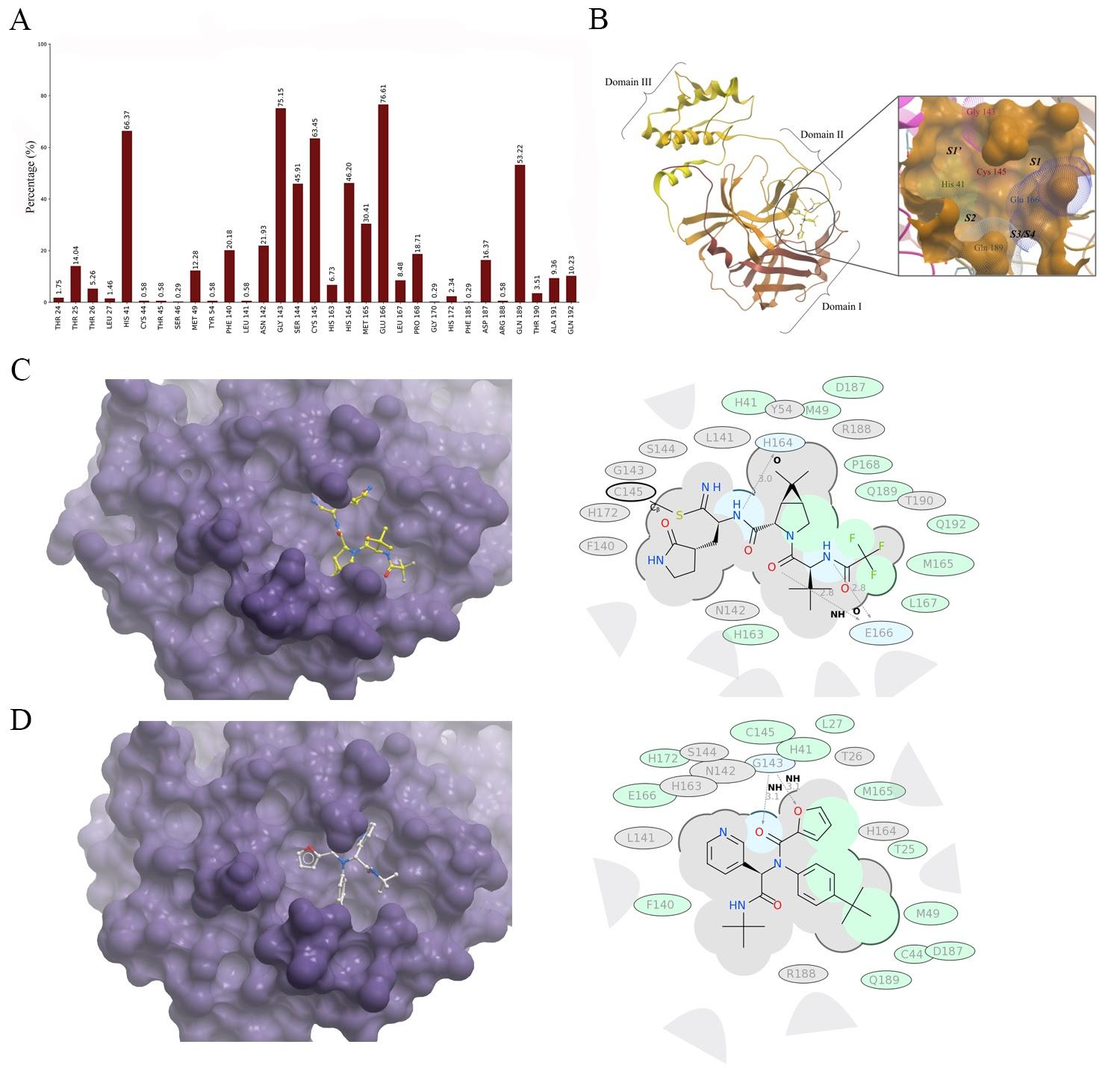
**

**Figure S1: (A)** Distribution of interacting amino acids across analyzed structures. **(B)** Domains of Mpro with detailed visualization of catalytic site with their sub pockets. **(C)** Example of the crystal structure of covalently inhibited main protease with the schematic 2D interaction plot. **(D)** Example of the crystal structure of non-covalently inhibited main protease with the schematic 2D interaction plot.


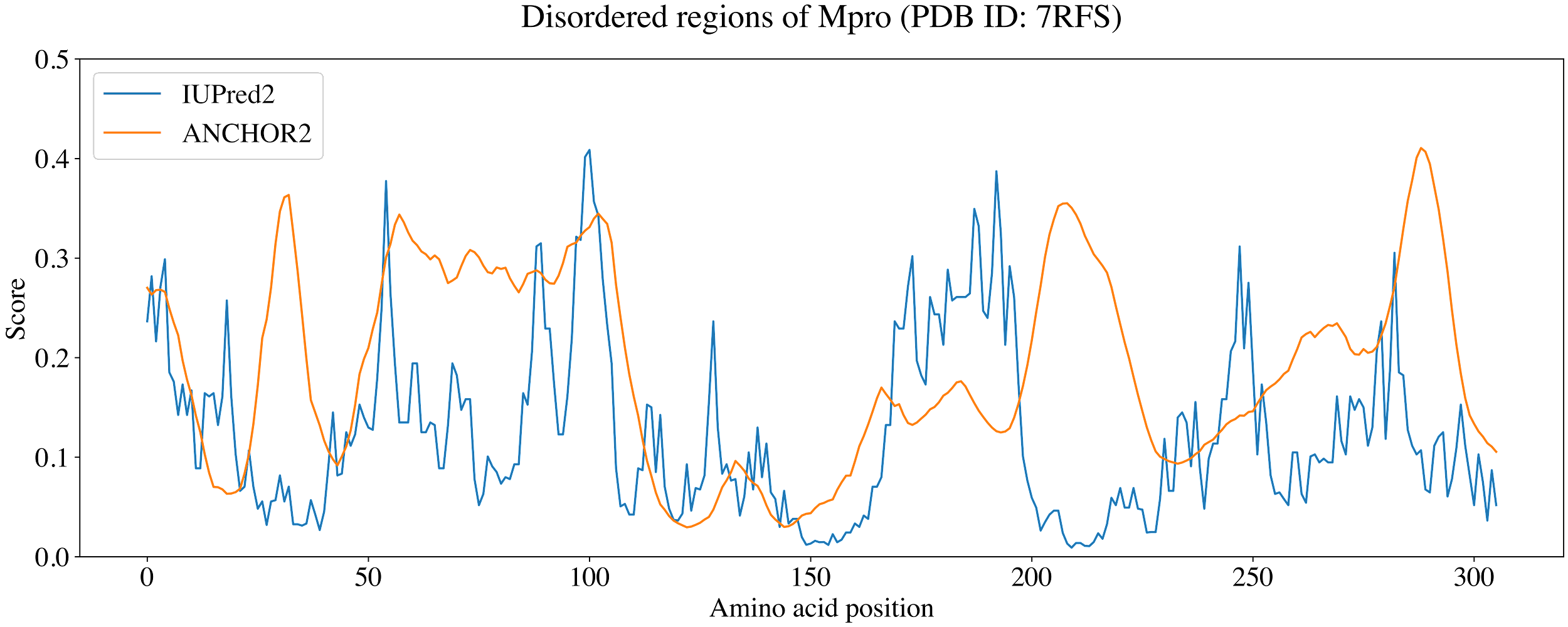


**Figure S2:** Disordered regions and disordered binding regions in the structure of the main protease identified by IUPred2 (long) and ANCHOR2 algorithms respectively.


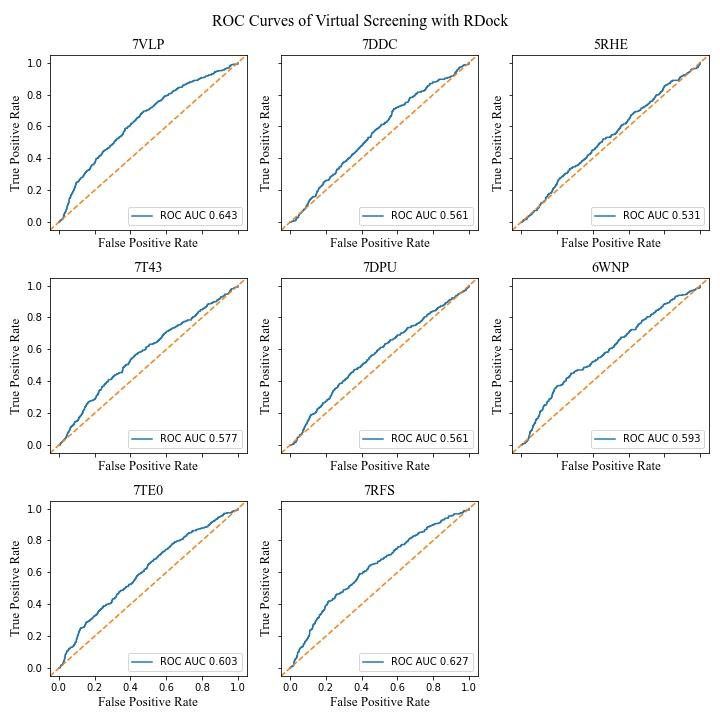


**Figure S3:** ROC curves with AUC values of virtual screening done by RDock docking software.


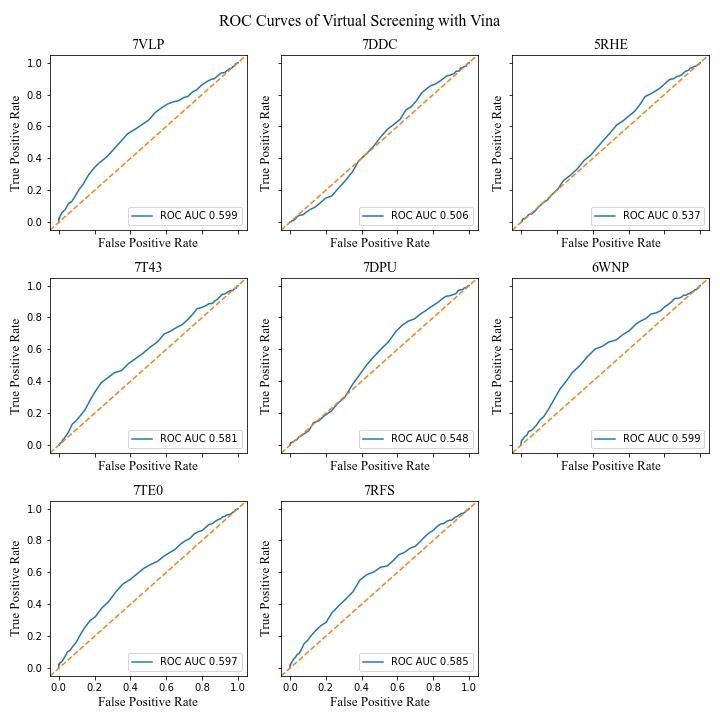


**Figure S4:** ROC curves with AUC values of virtual screening done by AutoDock Vina docking software.

**
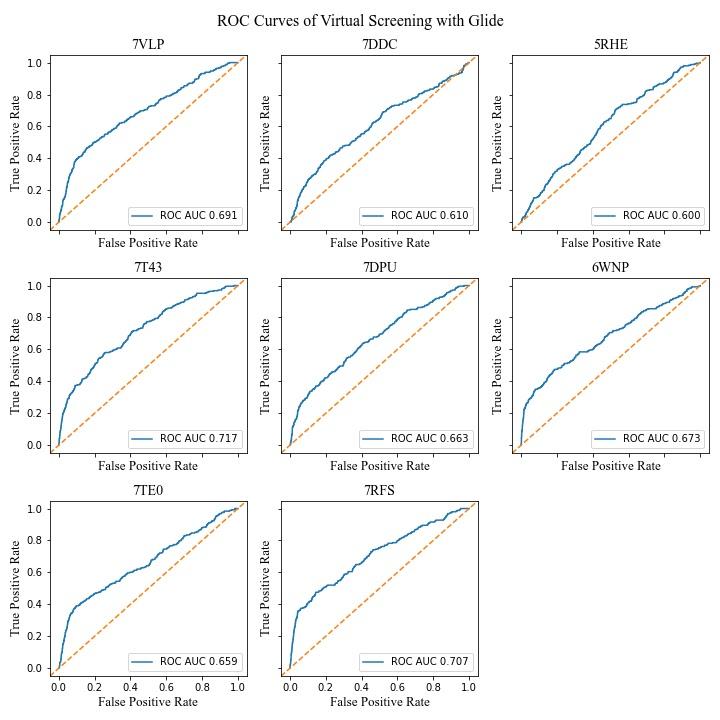
**

**Figure S5:** ROC curves with AUC values of virtual screening done by Glide docking software.


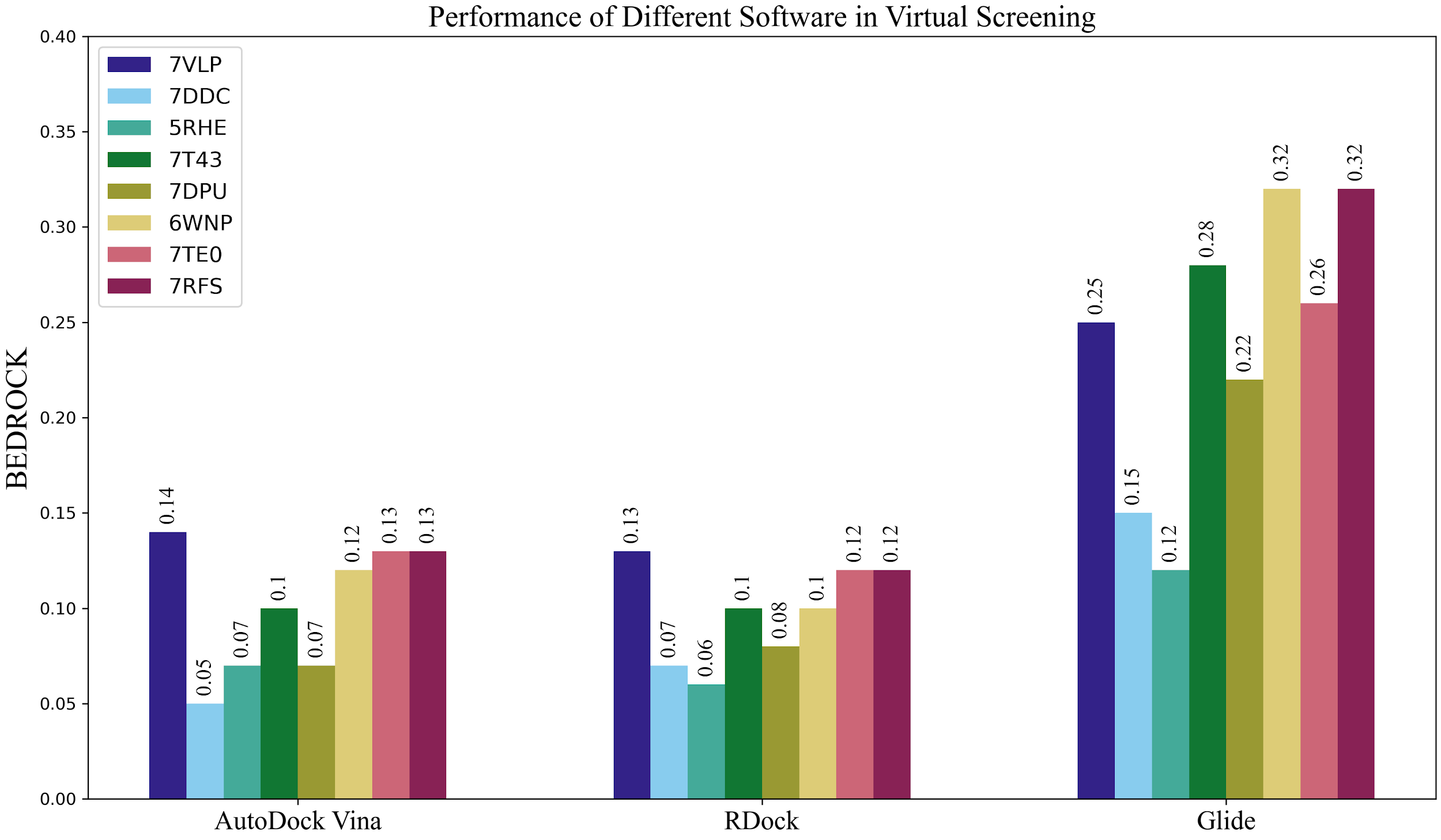


**Figure S6:** The performance of virtual screenings evaluated by the BEDROCK metric for each software.

**
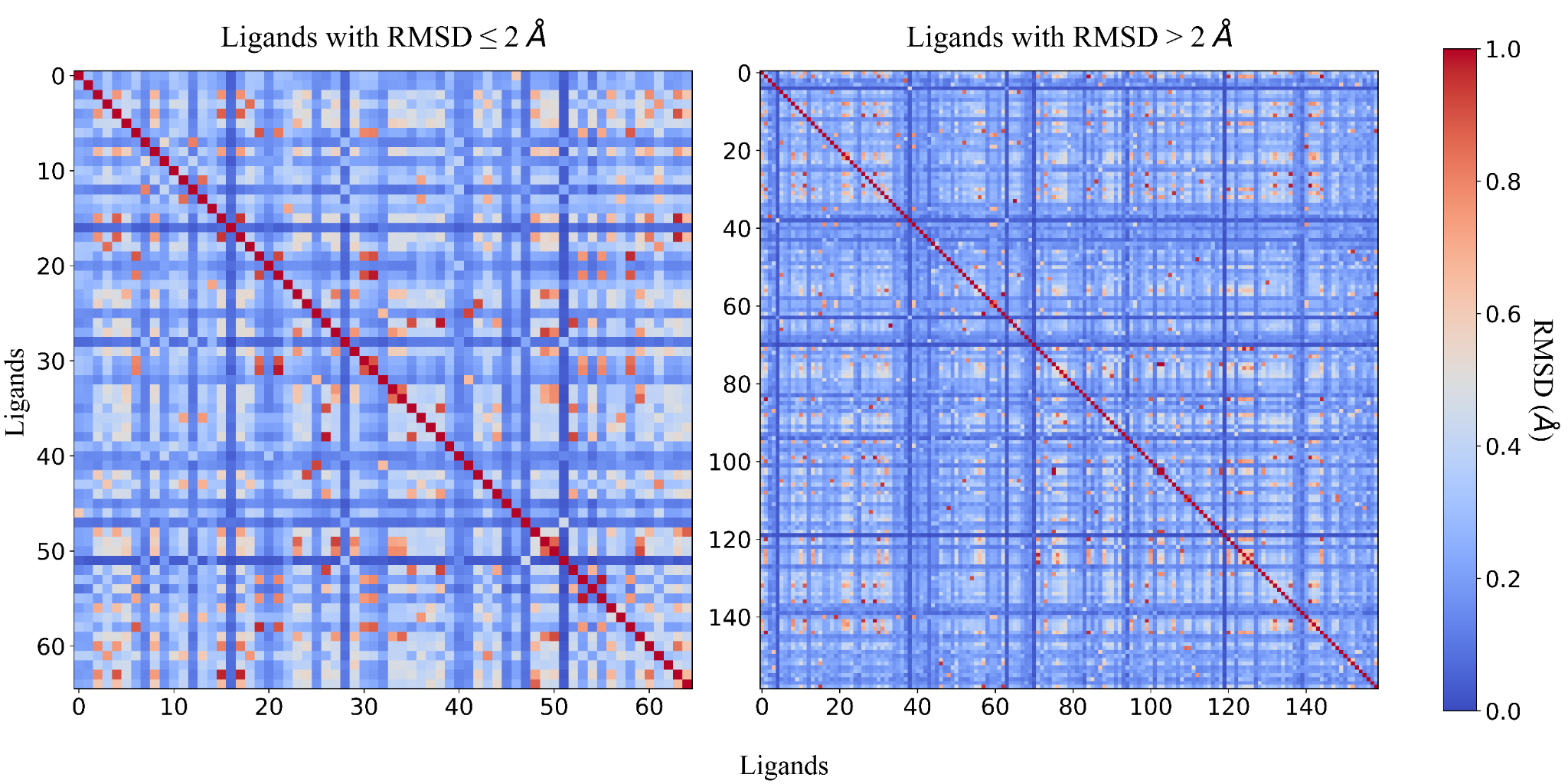
**

**Figure S7:** Tanimoto similarity heatmaps of molecules demonstrated RMSD less or equal to 2 Å and higher then 2 Å during screening with Glide and 7RFS structure.

| **Cluster N** | **Number of Unique Ligands** | **PDB ID of Representative Structure** | **Mean Tanimoto Distance in a Cluster** | **Average Molecular Weight (g/mol)** | **Average Number of Rotatable Bonds** |
| --- | --- | --- | --- | --- | --- |
| 1 | 1 | 7DDC  (lig: H3F) | 1 | 463.5 | 9 |
| 2 | 1 | 7DPU  (lig: HER) | 1 | 332.26 | 2 |
| 3 | 47 | 7T43  (lig: FN2) | 0.604 | 535.18 | 11.3 |
| 4 | 34 | 7RFS  (lig: 4WI) | 0.507 | 509.56 | 10.35 |
| 5 | 129 | 5RHE  (lig: UPD) | 0.199 | 294.43 | 3.87 |
| 6 | 40 | 7TE0  (lig: 4WI) | 0.422 | 479.05 | 8.3 |
| 7 | 2 | 7VLP  (lig: 4WI) | 0.407 | 547.85 | 10 |
| 8 | 58 | 6WNP  (lig: U5G) | 0.349 | 474.19 | 8.26 |

**Table S1:** Table summarizes the analysis of the structural diversity of the bound ligands within each identified cluster: mean Tanimoto distance, average molecular weight and average number of rotatable bonds of ligands within each cluster.

| **Amino acid residues** | **Mean (Å)** | **Max (Å)** |
| --- | --- | --- |
| The amino acid residues with the highest levels of fluctuation | | |
| Gly71 | 0.592 | 0.683 |
| Asp153 | 0.504 | 0.853 |
| Thr190 | 0.551 | 0.697 |
| Gly195 | 0.744 | 0.905 |
| Asn221 | 0.666 | 0.754 |
| Met276 | 0.907 | 1.17 |
| The amino acid residues with the lowest levels of fluctuation | | |
| His41 | 0.129 | 0.137 |
| Gly143 | 0.114 | 0.130 |
| Cys145 | 0.063 | 0.065 |
| The amino acid residues with relatively high mobility | | |
| Glu166 | 0.236 | 0.264 |
| Gln189 | 0.484 | 0.530 |

**Table S2:** Average and maximum values of fluctuations of important amino acids derived from the normal-mode analysis.
